# Complementing the pulp proteome via sampling with a picosecond infrared laser (PIRL)

**DOI:** 10.1007/s00784-021-03962-0

**Published:** 2021-05-12

**Authors:** Yaghoup Feridouni Khamaneh, Parnian Kiani, R. J. Dwayne Miller, Hartmut Schlüter, Reinhard E. Friedrich

**Affiliations:** 1 Department of Oral and Maxillofacial Surgery, University Medical Center Hamburg-Eppendorf, University of Hamburg, Hamburg, Germany; 2 Institute of Clinical Chemistry and Laboratory Medicine, University Medical Center Hamburg-Eppendorf, University of Hamburg, Hamburg, Germany; 3 Department of Periodontics, Preventive and Restorative Dentistry, University Medical Center Hamburg-Eppendorf, University of Hamburg, Hamburg, Germany; 4 DMD, Dental Clinic Zahnvitalis, Julius-Vosseler-Str. 42, D-22527 Hamburg, Germany; 5 Department of Chemistry, Lash Miller Chemical Laboratories, University of Toronto, 80 St. George Street, Toronto, ON M5S 3H6 Canada

**Keywords:** Picosecond infrared laser, PIRL, Mass spectrometry, Dental pulp, Proteomics, Proteome

## Abstract

**Objectives:**

The aim of this investigation was the detailed analysis of the human pulp proteome using the new picosecond infrared laser (PIRL)-based sampling technique, which is based on a completely different mechanism compared to mechanical sampling. Proteome analysis of healthy pulp can provide data to define changes in the proteome associated with dental disease.

**Material and methods:**

Immediately after extraction of the entire, undamaged tooth, 15 wisdom teeth were deep frozen in liquid nitrogen and preserved at −80°C. Teeth were crushed, and the excised frozen pulps were conditioned for further analysis. The pulps were sampled using PIRL, and the aspirates digested with trypsin and analyzed with mass spectrometry. Pulp proteins were categorized according to their gene ontology terminus. Proteins identified exclusively in this study were searched in the Human Protein Atlas (HPA) for gaining information about the main known localization and function.

**Results:**

A total of 1348 proteins were identified in this study. The comparison with prior studies showed a match of 72%. Twenty-eight percent of the proteins were identified exclusively in this study. Considering HPA, almost half of these proteins were assigned to tissues that could be pulp specific.

**Conclusion:**

PIRL is releasing proteins from the dental pulp which are not dissolved by conventional sampling techniques.

Clinical Relevance

The presented data extend current knowledge on dental pulp proteomics in healthy teeth and can serve as a reference for studies on pulp proteomics in dental disease.

**Supplementary Information:**

The online version contains supplementary material available at 10.1007/s00784-021-03962-0.

## Introduction

Many cellular functions of the tooth are concentrated in the dental pulp [1, 2]. The proteome represents the current functional status of a tissue. Knowledge of the dental pulp proteome of healthy teeth can serve as the basis for interpreting pathological changes in this organ. However, so far, there are only a few studies dealing with the proteome analysis of dental pulp. The studies differ from each other in the total number and characteristics of the proteins [3–7]. The differences in the number and composition of the identified dental pulp proteins from previous studies are in part due to different methods of determination as well as the presence of blood proteins, which can reduce the number of identifiable proteins because of the suppression effect in mass spectrometry (MS), which is in general a successful technique for protein analysis [8–12]. Prior to the identification of proteomes via MS, optimal sample preparation is required [13, 14]. The sampling parameters have an impact on the results, for example, due to latency times between the comminution of the sample and the analysis. The picosecond infrared laser (PIRL) has been introduced in sampling of biological tissues [15–19]. By irradiation with PIRL, tissue is immediately converted in a tissue aerosol by cold vaporization. The ultra-short pulsed, high-energy irradiation on the individual cell enables very fast cold vaporization of the tissue thereby yielding a better homogenate compared to mechanical sampling. The cold vaporization of the tissue induced by PIRL is very soft and therefore labile biomolecules like proteins remain intact [15, 19]. In the tissue aerosol all molecules of the tissue are present and dissolved in water droplets smaller than 10 μm. Subsequent proteolytic digestion of the proteins in the condensed tissue aerosol followed by tandem MS (MS/MS) of the proteolytic peptides is providing a significant larger yield of identified proteins compared to classical mechanical sampling and homogenization techniques. Therefore, this method was chosen for the proteome analysis of human dental pulp.

The aim of this study was to widen the database for the proteome composition of healthy human pulp, which enables protein biomarkers to be determined for the diagnostic-therapeutic approach of dental diseases, such as inflammation of the pulp. Sampling of the tooth pulp with PIRL prior to mass spectrometric proteome analysis is a major new approach promising additional insights into the tooth pulp proteome. In addition, we classified the proteins into groups so that a structural overview of the pulp’s protein composition can be obtained by assigning gene ontology terms (annotation) to the individual proteins, namely molecular functions, the biological processes, and the cellular components.

## Methods

### Sample preparation

A total of 15 healthy wisdom teeth from 6 patients (2 men and 4 women) aged 19–42 years were studied. The teeth were removed under general anesthesia in the Department of Oral and Maxillofacial Surgery of the University Hospital Hamburg-Eppendorf without complications, immediately cleaned with NaCl of blood and saliva, transferred to 15 ml centrifuge tubes and frozen in liquid nitrogen. One tooth was stored per centrifuge tube. The teeth were stored at −80°C until the pulps were extracted from the teeth. For this purpose, the teeth were crushed with a hammer maintaining sterile surgical conditions to avoid any contamination of samples. The exposed pulp was removed with sterile forceps, weighed, and transferred to an Eppendorf tube and stored again at −80°C until processed with the PIRL. In average, the weight of each sample was 20 mg.

The laser system PIRL was used for dissolving the samples. The laser system was equipped with a water-cooled copper block connected to a temperature controller. The system always was keeping constant the temperature of the tissue at −10°C. The copper block was in a self-made, tightly sealed chamber. The block had a streamlined-shaped interior. The dimensions of the block are designed to produce an aerodynamic, constant laminar air flow by a diaphragm pump generating a negative pressure in the interior. The chamber has a window for the inlet of the laser beam and two further openings for the supply and removal of air. The chamber was connected via one of these openings through a teflon tube to a collecting trap lined with glass fiber filter (Hahnemühle, Dassel, Germany; technical data: grade FP GF 50, weight: 56 g/qm, thickness: 0.29 mm and raw material: borosilicate glass without binder). This device was connected to a vacuum pump via another teflon tube. The ablation cloud (“plume”) formed during the irradiation was sucked off and collected in the clamped device with a glass fiber filter.

Each single pulp sample was placed individually on the copper block under the laser beam inlet window and irradiated with the maximum laser energy density per area of 1.5 J/cm^2^. The irradiation took place until the complete dissolution of the samples. The irradiated tissue is transferred into the gas phase together with the dispersed water molecules contained in the tissue. The resulting aerosol is collected using the negative pressure in the trap. The vacuum vaporizes the water from the aerosol. Thus, the collected biomolecules are pulverized and fixed on the glass fiber filter. The glass fiber filter was transferred to a new Eppendorf tube and stored at −80°C until assayed. Between each sample, all objects (chamber, suction tube, filter element) were thoroughly cleaned with water and isopropanol and disinfected and a new glass fiber filter was clamped in the trap.

### Tryptic digestion of the pulp proteins

For solubilization of tissue aerosol condensate, 500 μl sodium deoxycholate (SDC) buffer (1% SDC, 0.1 M triethylammonium bicarbonate (TEAB)) was added into each reaction vial containing the glass fiber filter.

The vials were centrifuged for 5 min at 10,000×$\overrightarrow{g}$. The supernatants were transferred into new vials, the samples were then treated for 30 s in the ultrasonic homogenizer at 25% power and then incubated at 99°C for 30 min. They were again centrifuged for 5 min at 10,000×$\overrightarrow{g}$. The supernatant was transferred to a centrifuge filter (cut-off 10 kDa, Amicon, Merck, Darmstadt, Germany). The pellet was later processed further. The supernatant in the centrifuge filter was centrifuged at 14,000×$\overrightarrow{g}$ for 30 min, and the filtrate was disposed.

Four hundred microliters of urea (8 M) were then added to the vial with the pellet from the SDC processing and centrifuged for 5 min at 10,000×$\overrightarrow{g}$. The supernatant was transferred to the same centrifuge filter containing the proteins from SDC extraction and centrifuged at 14,000×$\overrightarrow{g}$ for 30 min.

Five hundred microliters of urea (6 M) were added to the centrifuge filter with the extracted proteins. The proteins were concentrated by centrifuging at 14,000×$\overrightarrow{g}$ for 30 min. The filtrate was disposed. This step was repeated another three times to completely remove MS-incompatible SDC buffer. The protein concentration was measured with the Pierce BCA Protein Assay Kit (Pierce, Rockford, IL, USA) using BSA as the standard and according to the manufacturer’s instructions. Thirty micrograms of protein were used for tryptic digestion. The disulfide bridges of the proteins were reduced with 1 μl dithiothreitol (DTT) (100 mM, dissolved in ammonium bicarbonate (AmBiCa), pH = 8.3) at 56°C and 15 min incubation time. Subsequently, the sulfide ions were alkylated with 1.3 μl iodoacetamide (IAA) (300 mM, dissolved in AmBiCa, pH = 8.3) at room temperature in the dark for 30 min incubation time. Then, 425 μl of AmBiCa (100 mM) was added to obtain a basic medium. Tryptic digestion was carried out at 37°C for 16 h with 2.5 μg trypsin (*c* = 0.25 μg/μl). Finally, it was centrifuged at 14,000×$\overrightarrow{g}$ for 30 min. The filtrate contained tryptic-digested peptides. The solvent was concentrated in vacuum to dryness.

### Liquid chromatography mass spectrometry analysis

Each of the tryptic peptides of the 15 samples was dissolved in 10 μl 0.1% formic acid and 5 μl were analyzed with a liquid-chromatography coupled to a tandem mass spectrometry system (LC-MS/MS). For this purpose, the peptides were separated in a nanoUPLC system (UltiMate 3000 Rapid Separation Liquid Chromatography (RSLC), Dionex, Thermo Scientific, Waltham, Mass., USA) coupled with electrospray ionization (ESI) on a hybrid quadrupole orbital mass spectrometer (Orbitrap QExactive, Thermo Scientific) and measured. The samples were injected at a flow rate of 5 μl/min with a 5% buffer B (0.1% formic acid (FA) in acetonitrile (ACN)) on a reversed-phase pre-column (Acclaim PepMap μ-precolumn, C18, 300 μm × 5 mm, 5 μm, 100×, Thermo Scientific). The pre-column was first washed for 5 min at a flow rate of 3 μl/min and then the peptides were eluted at a flow rate of 350 nl/min on a reversed-phase separation column (Acclaim PepMap 100, C18, 75 μm × 250 mm, 2 μm, 100 Å, Thermo Scientific). The peptides were eluted with a binary buffer system buffer A (0.1% FA in high-performance liquid chromatography (HPLC)-H_2_O) and buffer B (0.1% FA in ACN) and a gradient of 5–32% buffer B in 120 min with a total chromatography time of 160 min.

The spray was produced by a fused silica emitter (inner diameter: 10 μm, New Objective, Woburn, USA) at a capillary voltage of 1650 V. The mass spectrometric measurement was performed in the positive ion mode, in the data-dependent-acquisition (DDA) mode with the TopN mode 12. At the first MS level (MS-1), the precursor ions were measured in a scan range of 400–1220 *m*/*z* with a resolution of the Orbitraps of QExactive of 70,000 fill width at half maximum (FWHM) at 200 *m*/*z*. The maximum injection time was 120 ms, and the automatic gain control (AGC) targets 1 × 10^6^. On the second MS level (MS-2), the following parameters for fragmenting the precursor ions were selected as selection criteria: the intensity threshold value with 1 × 10^5^ in an isolation window of 2 *m*/*z*. The fragmentation took place in a resolution of 17,500 FWHM at 200 *m*/*z*. For fragmentation, ions with the charge 2+ to 7+ were selected. The maximum injection time was 60 ms, the AGC target 5 × 10^5^, and the minimum AGC targets 6 × 10^3^. The fragment spectra were recorded in a scan range of 200–2000 *m*/*z* at a scan rate of 1 s.

### LC-MS/MS data analysis

The raw data of the LC-MS/MS analysis were processed with the MaxQuant software version 1.5.8.3 (Max-Planck Institute for Biochemistry, Planegg, Germany). The test results were compared with the data from the human database (Uniprot, 2019; https://www.uniprot.org/proteomes/) as well as contaminant database with a false discovery rate (FDR) value <0.01 at the protein and peptide level and modifications. As a protease specification, trypsin was chosen. The following parameters were selected for MaxQuant: two interfaces for arginine and lysine were considered as faulty interfaces. For the mass tolerance for precursor at MS-1, 10 ppm was chosen, and for fragmentation at MS-2, 0.02 Da. The following modifications were chosen as variable modifications: oxidation on methionine and acetylation on protein N-terminus. Subsequently, as a fixed modification, carbamidomethylation was selected on cysteine. For identification, only proteins with unique peptides were used (number of unique peptides per protein ≥1).

In order to study the composition of molecular function, cellular components, and biological processes, the proteins were tested on the website PANTHER [20] (Protein ANalysis THrough Evolutionary Relationships) Classification System. For protein classification, the database of *Homo sapiens* was queried. Web application BioVenn [21] was used to compare the results with the literature and the creation of Venn diagrams.

For further analysis of the protein list, the platforms Uniprot [22], Open Targets Platform [23], and The Human Protein Atlas (HPA) [24] were used.

The platform HPA was used exclusively for the 382 proteins that were identified only in this study.

## Results

### General characteristics of pulp proteome

In the analysis of 15 wisdom teeth, 1077 protein families (unique peptides ≥1) were identified. The Gene Ontology (GO) classifications (GO annotations: molecular functions, cellular components, and biological processes) were examined in more detail. For this purpose, the protein list obtained from the LC-MS/MS data of this study was classified and compared with the protein lists from Eckhardt et al. [6] and Eckhard et al. [7] using the PANTHER database [20] (see Figs. 2, 4, and 6). In Table 1 (in the supplement), all protein families are listed. The results of the GO analysis are giving a very rough overview only about the proteins and their assigned molecular functions (Fig. 2), localizations (Fig. 4), and participation in cellular physiological processes (Fig. 6). Since it is only a rough measure and should not be mixed with a precise quantification, the given results in the Figs. 2, 4, and 6 must be interpreted as not significantly different. This interpretation is in line with the results shown in Fig. 7. There is an overlap of proteins, which have been identified in all studies, which is similar in relationship to the number of proteins, identified in the individual studies. All studies represent proteins which cover all aspects of cell functions, organization, and cell physiology, which is necessary for the cells in the tooth pulp and its physiological functions. The abundance of the proteins which can be assigned to the different individual GO terms is covering a broad range. This is the reason why the distribution of the identified proteins to the different GO terms is not depending on the total number of identified proteins.

**Fig. 1:**
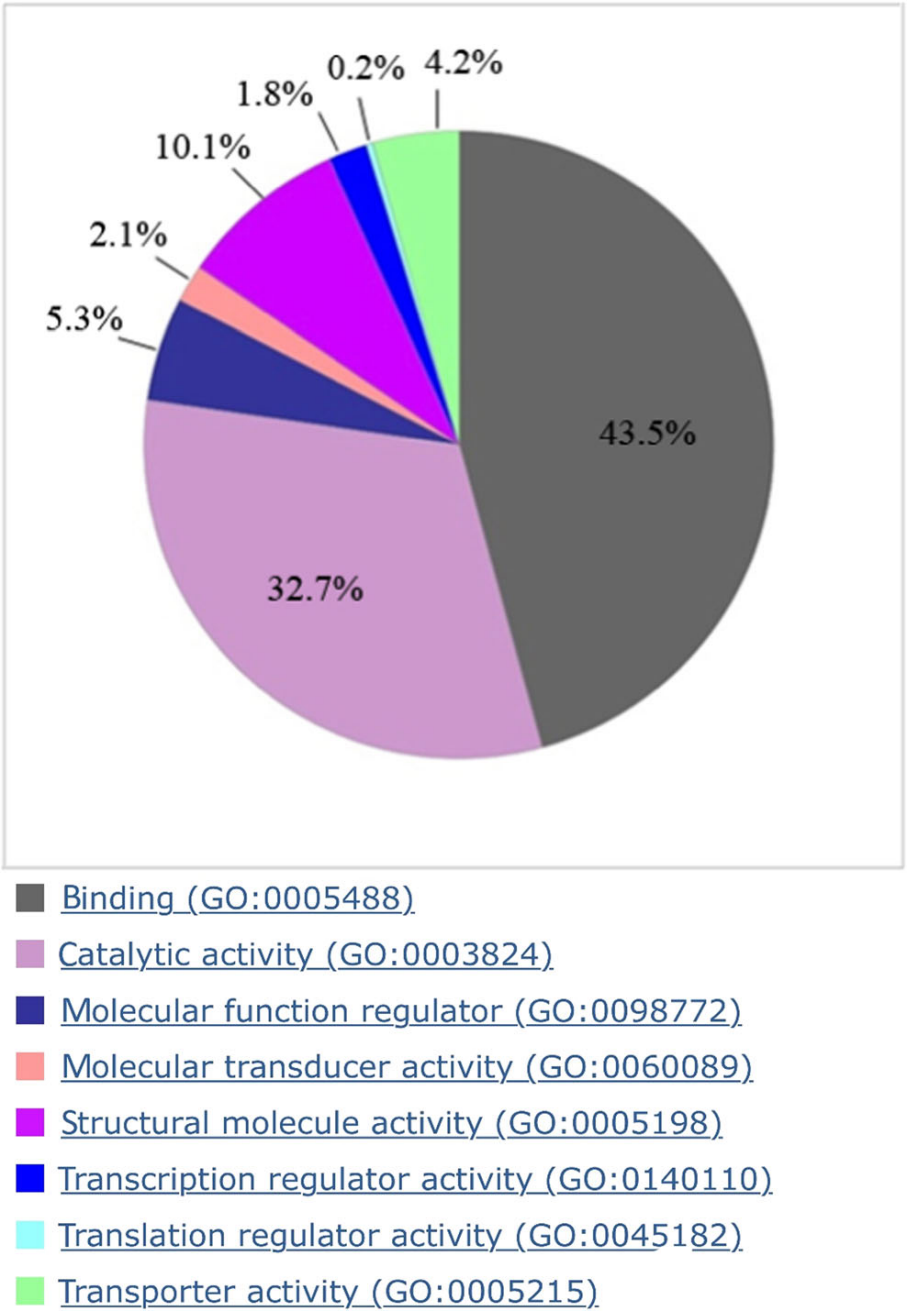
Pie chart of the molecular functions with explanation of the respective GO classification

Examination of molecular functions of the identified proteins revealed that 43.5% of the proteins are subdivided into the category binding (GO: 0005488) (Fig. 1). The bindings in turn were subdivided into the subclasses: 42.6% protein binding (GO: 0005515), 25.1% heterocyclic binding (GO: 1901363), 10.9% ion binding (GO: 0043167), 6.1% small molecule binding (GO: 0036094), 4.4% organic cyclic compound binding (GO: 0097159), 4.2% protein-containing complex binding (GO: 0044877), 2.0% drug binding (GO: 0008144), 1.6% amide binding (GO: 0033218), 1.3% chromatin binding (GO: 0003682), 0.8% cofactor binding (GO: 0048037), 0.7% carbohydrate derivative binding (GO: 0097367), 0.3% lipid binding (GO: 0008289), and 0.1% neurotransmitter binding (GO: 0042165).

A further subdivision of the molecular function after the binding follows with 32.7% the catalytic activities (GO: 0003824), which are divided into the following groups: 47.1% hydrolysis activity (GO: 0016787), 16.8% oxidoreductase activity (GO: 0016491), 13.3% transferase activity (GO: 0016740), 7.8% lyase activity (GO: 0016829), 4.4% ligase activity (GO: 0016874), 18.3% protein activity on catalytic activities (GO: 0140096), 2.5% intramolecular transferase activity (GO: 0016866), and 1.7% further catalytic activity (GO: 0140098, GO: 0140097). This is followed by further molecular function with 10.1% structuring molecule activity (GO: 0005198), 5.3% molecular function regulator (GO: 0098772), 4.2% transporter activity (GO: 0005215), 2.1% molecular transducer activity (GO: 0060089), 1.8% transcriptional regulator activity (GO: 0140110), and 0.2% translational regulatory activity (GO: 0045182).

**Fig. 2:**
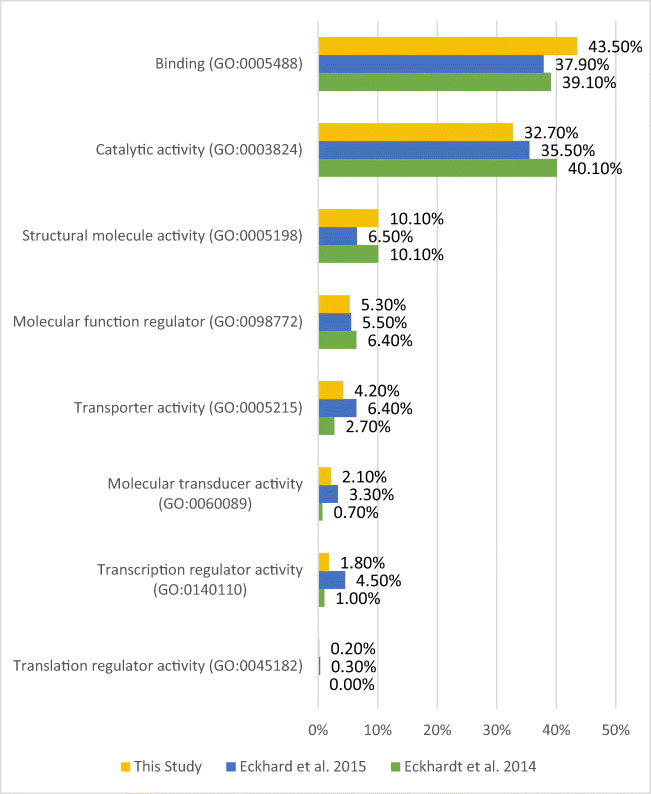
Percentage comparison of molecular function with literature

Figure 2 compares the percentage composition of functions with the results of the respective literature.

### Cellular components

**Fig. 3:**
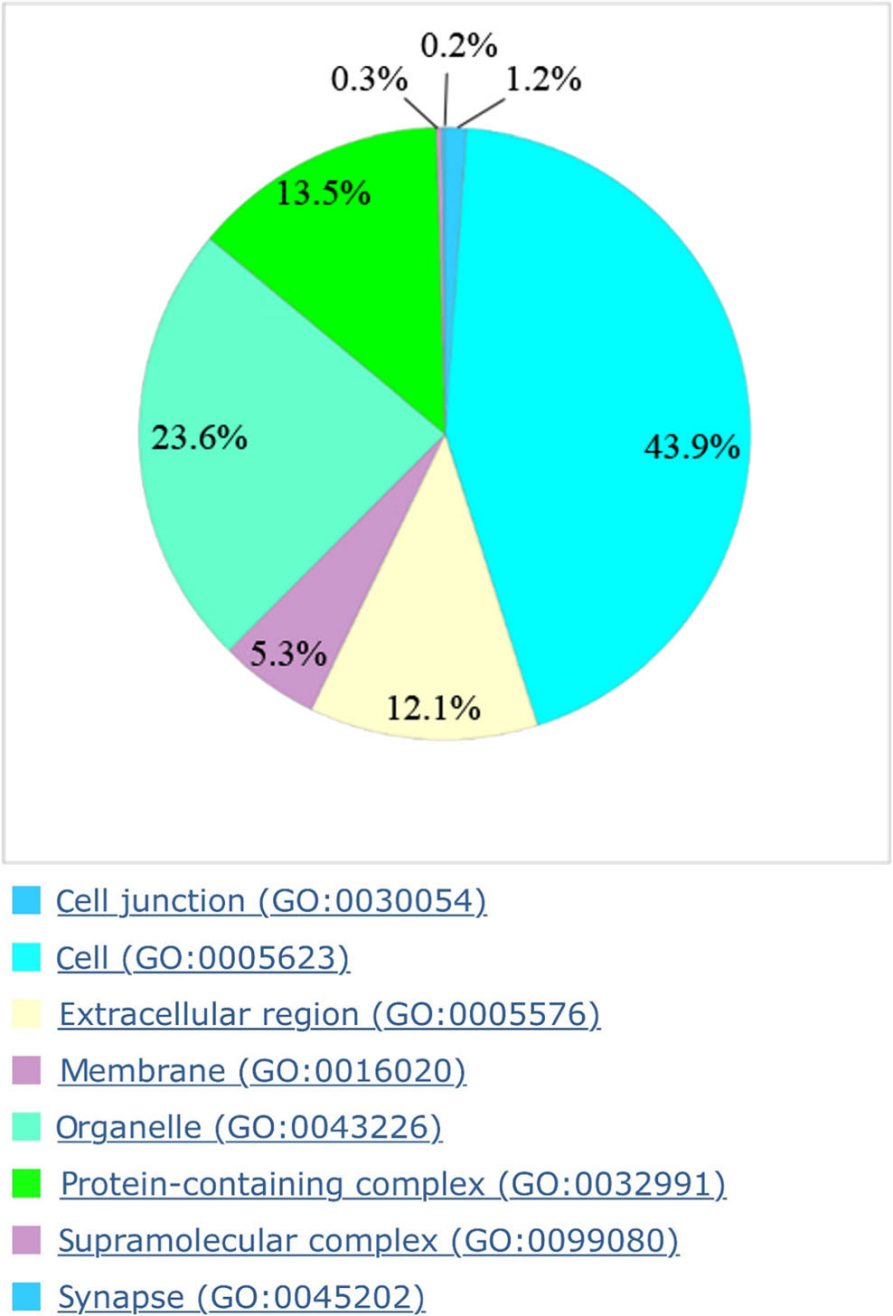
Pie chart of the cellular components with explanation of the respective GO classification

**Fig. 4:**
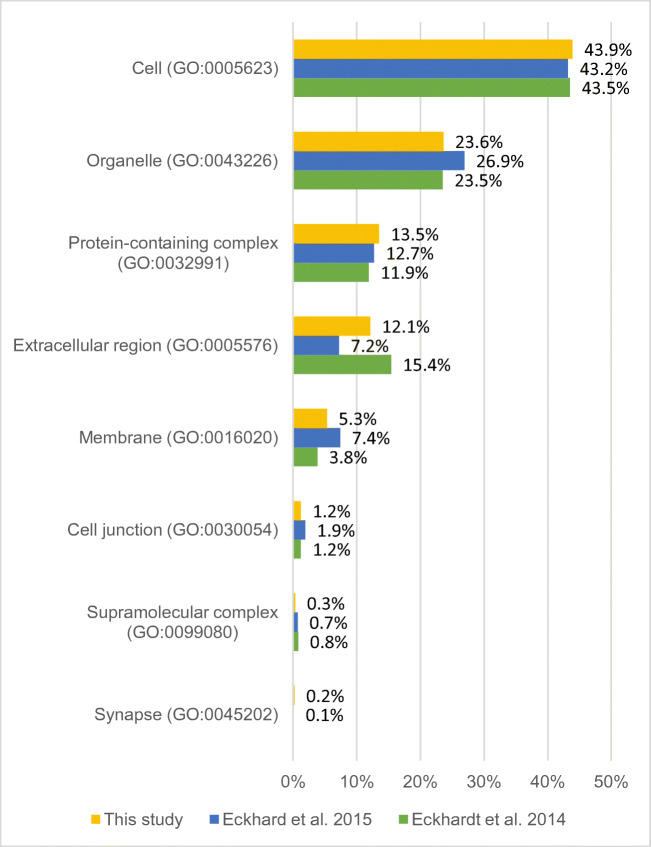
Percentage comparison of cellular components of the proteins identified in pulp tissue in this study with prior studies

Figure 3 shows the cellular components of pulp tissue in a pie chart consisting of 43.9% cell parts (GO: 0005623), 23.6% organelles (GO: 0043226), 13.5% protein-containing complexes (GO: 0032991), 12.1% extracellular region (GO: 0005576), 5.3% membrane (GO: 0016020), 1.2% cell junctions (GO: 0030054), 0.3% supramolecular complex (GO: 0099080), and 0.2% synapses (GO: 0045202). Figure 4 compares the percentage composition of the cellular components with the results of the respective literature.

### Biological processes of the proteins identified in pulp tissue

**Fig. 5:**
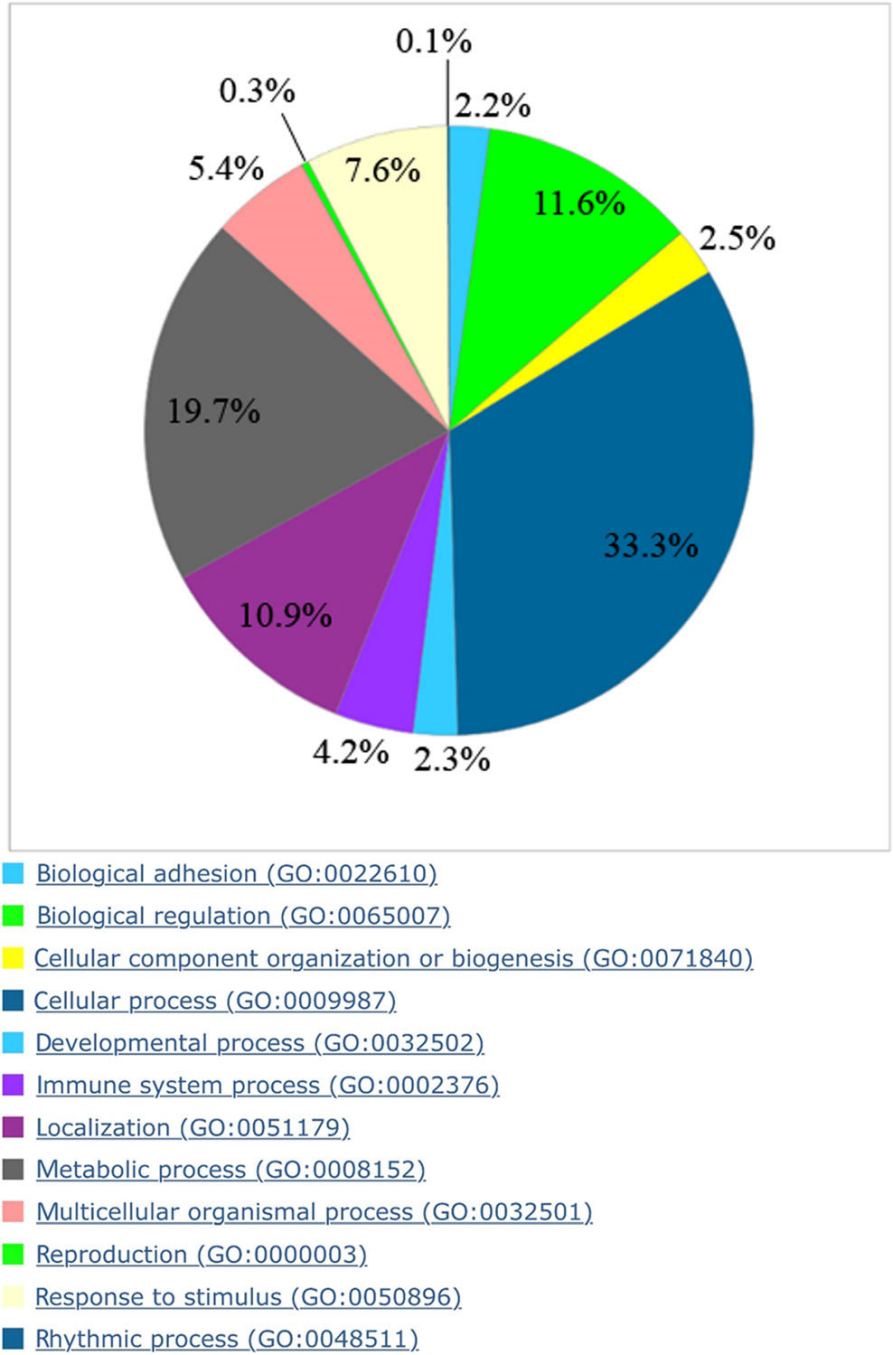
Pie chart of the biological processes of the proteins identified in pulp tissue with explanation of the respective GO classification

**Fig. 6:**
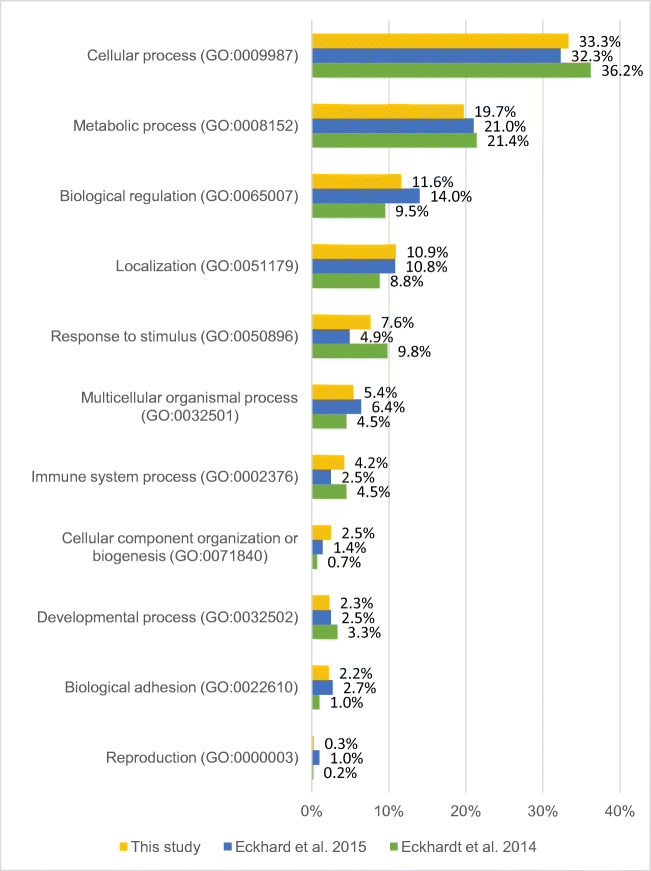
Percentage comparison of biological processes of the proteins identified in pulp tissue prior studies

Figure 5 shows the biological processes in a pie chart. The biological processes are composed of 33.3% cellular processes (GO: 0009987), 19.7% metabolic processes (GO: 0008152), 11.6% biological regulation (GO: 0065007), 10.9% localization (GO: 0051179), 7.6% response to stimulus (GO: 0050896), 5.4% multicellular organismal process (GO: 0032501), 4.2% immune system process (GO: 0002376), 2.5% cellular organism component or biogenesis (GO: 0071840), 2.3% developmental process (GO: 0032502), 2.2% biological adhesion (GO: 0022610), 0.3% reproduction (GO: 0000003), and 0.1% rhythmic process (GO: 0048511). Figure 6 compares the percentage composition of biological processes with the results of the respective literature.

### Results in protein families and protein list of the proteins identified in pulp tissue in comparison with the literature

In this study, 1077 protein families and 1348 proteins have been identified (see Table 1). Identified proteins of this study compared with the study of U. Eckhardt et al. from 2014 and A. Eckhard et al. from 2015.

**Fig. 7:**
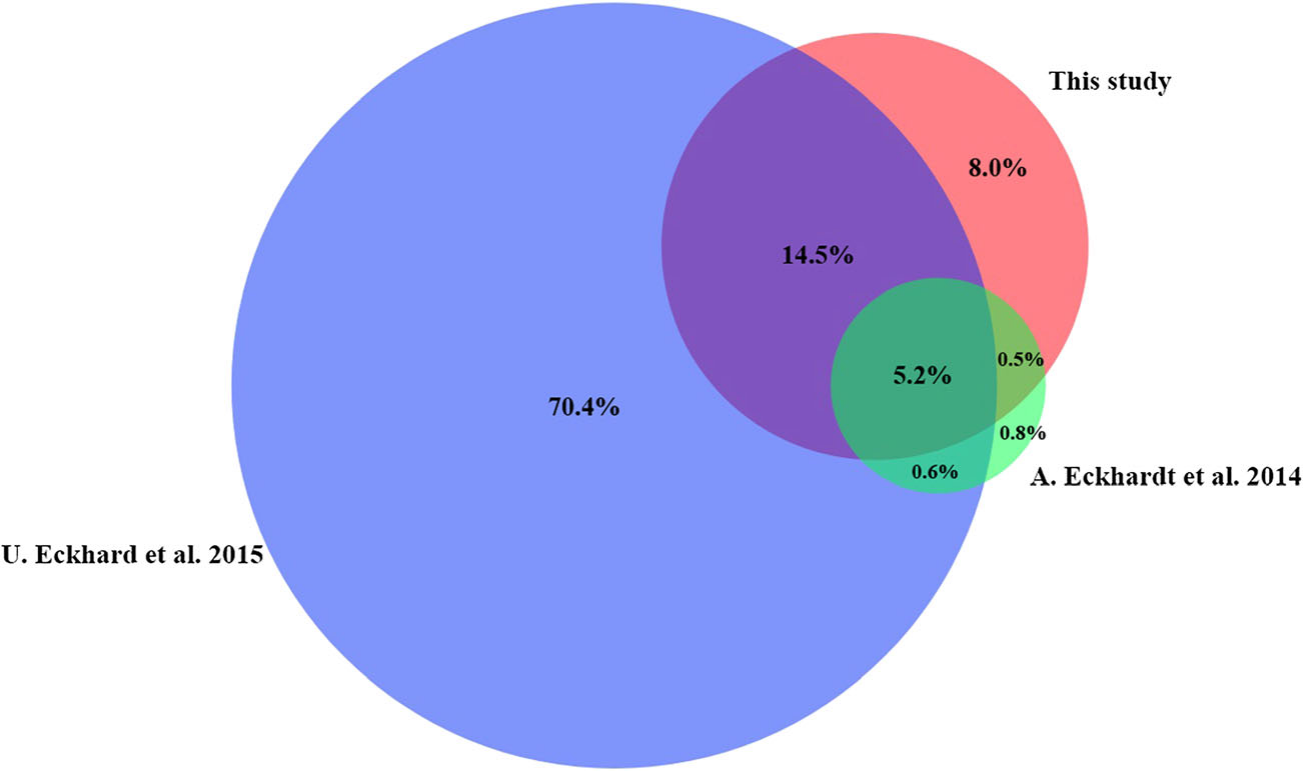
Venn diagram: percentage comparison of the identified proteins from this study with previous studies: Eckhardt et al. [6] as well as Eckhard et al. [7].

The Venn diagram shows the percentage distribution of proteins in comparison with the studies by Eckhardt et al. [6] and Eckhard et al. [7] (Fig. 7).

## Discussion

A total of 1077 protein families and 1348 proteins of the pulp were identified in this study. Compared to conventional mechanical homogenization methods, e.g., in Eckhardt et al. [6] and Eckhard et al. [7], homogenization and extraction were performed in this study within a shorter period of time by irradiation with an innovative infrared laser system. The investigation identified proteins which were previously unknown as pulp components.

Table 3 shows a complete listing of all pulp proteins identified so far, based on the work of this study, Eckhardt et al. [6] as well as Eckhard et al. [7].

Kwiatkowski et al.’s [19] analysis of pancreatic and tonsil tissues revealed that, compared to conventional mechanical tissue homogenization techniques, PIRL homogenization provides a significant higher number of intact protein species analyzed by 2D gel electrophoresis and high-resolution MS. The fine homogenate had a high quality due to the absence of particles such as cell debris. Due to the particle presence, the homogenate from the mechanical procedure requires further centrifugation steps, which in turn require more working time. It also shows that not all parts of the tissue are successfully homogenized. Nevertheless, the results of this study show that PIRL can be used successfully in the homogenization of tooth pulp tissue and high numbers of proteins can be identified from the homogenates using tandem MS.

Comparing the proteomics results of this study with the results of the prior studies [6, 7], 72% of 1348 identified proteins matches with their results. The remaining 28% were identified exclusively in this study. These proteins will be discussed in more detail in the next sections.

However, the differences between presented study results and those of previous reports are slight if the percentage of proteins is categorized according to “molecular function,” “biological processes,” and “cellular component” of all 1348 proteins (Figs. 2, 4, and 6).

The categorization is based on the website PANTHER and is a typical nomenclature for this classification [20]. Most pulp proteins functionally classified according to GO annotation are part of the groups “binding” (GO: 0005488) and “catalytic activities” (GO: 0003824).

The cellular component of the pulp consists mainly of cells (GO: 0005623). The main part of the biological processes of the identified pulpal proteins takes part in the cellular processes (GO: 0009987).

If procedural time of this study is compared with data of Eckhardt et al. [6] and Eckhard et al. [7], the current material analysis is carried out in about half the time (72 h vs. 36 h). Reducing the number of trials may minimize the associated contamination risks and sample loss.

A previous study on pulp proteome identified more proteins than in this study [7]. However, there are significant differences and goals between the two studies. Eckhard et al. (2015) have specifically modified the proteins to identify even less abundant proteins. On the one hand, this concept maximizes the throughput dynamics of protein identification. On the other hand, the procedure is very time consuming, costly, and cannot be used in routine examinations, which is among other characteristics an outlook of this study. Furthermore, the presented setting uses a very rapid procedure. The reaction of the subcellular comminution proceeds so quickly that degradation of the protein complexes in the samples is prevented. Therefore, the protein composition is very close to the in vivo state [19]. Indeed, previous study showed that the proteins remain intact due to the gentle irradiation with the PIRL. The rapid homogenization inhibits effectively intracellular enzymatic degradation [15] and artefactual modifications of proteins [19].

The characteristics of the PIRL-assisted MS analysis could be used to reevaluate protein functions, which currently are defined as biomarkers in dental diseases.

As an example, Ma et al. [25] classified 16 proteins that showed increased expression in carious teeth. Thirteen of these proteins (CCT2, TPM2, TALDO1, CAPZB, MYL9, HSP90AA1, TARDBP, CAPG, STMN1, APEH, HNRNPF, KRT9, and KRT10) are also featured in this study that have been performed on healthy, impacted wisdom teeth.

Jágr et al. [26] carried out a proteome analysis of human dental pulp, which investigated caries-free and carious teeth. Here, 12 proteins were identified that show both down and up regulations. The following 11 proteins are also present in this study (SERPINA1, HSPD1, ATP5B, PGK1, TPM1, VIM, ANXA2, ANXA5, PRDX1, APOH, and TF).

Another protein found in this study, alpha-2-macroglobulin, has been reported in a study examining the characteristics of inflammation in pulp tissue. There were statistically significant differences in the concentration of alpha-2-macroglobulin in moderate to severe inflammation compared to normal pulp tissue, as well as between mildly inflamed and moderately to severely inflamed pulps [27]. The increased concentration of the protease inhibitor in pulp inflammation indicates its role in the pathogenesis of inflammatory diseases of the pulp, e.g., a tooth decay or pulpitis.

In this study, 1077 protein families were identified. These 1077 protein families yield 1348 proteins, of which, according to Fig. 7, 382 proteins are found only in this analysis. If these proteins are classified, it is striking that 187 of these proteins belong to protein families, which were identified also in Eckhardt et al. [6] and Eckhard et al. [7]. The difference is that while only a few proteins from each protein family were identified in the studies mentioned, several proteins from a single protein family were identified in this study. An example is the protein family Histone H2A. Previous studies identified only 6 proteins from this family of proteins. With this method, the number of proteins of this protein family has increased to 15. By using the PIRLs more proteins of a well-known protein family could thus be identified.

A total of 181 protein families were identified exclusively in this study. Out of the 181 protein families, 195 proteins are derived. Of the 181 protein families, 170 each contain only one protein. When reviewing the 195 proteins, almost all proteins are classified as associated with common diseases. Only 4 proteins (CCDC124, IGLC6, PPIAL4D, and TRIM64B) are not classified as disease-associated according to the Open Targets Platform. In the literature, CCDC124 is mentioned in connection with cytokinesis and the associated midbody structure, a short-lived connection structure at the end of cell division [28]. IGLC6 belongs to immunoglobulins and is active in the field of immune response. There is no publication about PPIAL4D and TRIM64B function in the pulp until today. Further research is needed on these 4 proteins, especially regarding functions in the pulp.

For the remaining proteins, the following proteins are interesting from a dental perspective: high mobility group protein B1 (HMGB1), tissue inhibitor of metalloproteinase 2 (TIMP2), cluster of differentiation 59 (CD59), thymosin beta-10 (TMSB10), endoplasmic reticulum resident protein 29 (ERP29), sodium-dependent phosphate transporter 2 (SLC20A2), thymosin beta-4 (TMSB4X), platelet-derived growth factor receptor beta (PDGFRB), matrix extracellular phosphoglycoprotein (MEPE), family with sequence similarity 20, member C (FAM20C), small-ubiquitin-related modifiers 2 (SUMO2), small-ubiquitin-related modifiers 4 (SUMO4), and stathmin (STMN1).

The HMGB1 is a chromatin protein that causes inflammatory response and supports tissue repair. In one study, the expression and distribution of the protein in inflamed pulps were investigated. mRNA expression of HMGB1 was significantly increased probably indicating the inflammatory status of the organ. In addition, the protein is expected to play an important role in the recruitment of pulpal stem cells and to promote repair and regeneration of the pulp [29].

TIMP2 is upregulated in inflamed pulps [30].

Investigations concerning the complement system in inflammatory and regenerative processes in pulps, mentioned the protein CD59 as an inhibitor of the formation of the membrane attack complex. This complex is characteristic for its bacterial lytic activity and was used in studies to investigate bacterial contamination by *Streptococcus mutans* and *Streptococcus sanguinis*. Stimulated by fibroblasts of the pulp, the complex attacks bacteria. As a control study of the effectiveness of the membrane attack complex, a significant reduction of the complex was observed when inhibitor CD59 was added [31]. Further investigation of the protein CD59 should find out if it is assigned to other tasks. Here, it would be worthwhile to modify the protein to prevent the membrane attack complex from being blocked, so that the complex formation by fibroblasts of the pulp could better combat a bacterial load.

The mentioned proteins, like the proteins mentioned above, could be classified as protein biomarkers in the clinical study of pulps which show changes due to inflammatory diseases.

The following proteins are mainly active in the formation of different tooth structures.

The expression pattern and functions of the protein TMSB10 was studied during tooth development in lower first molars of mice. It was found that the protein was detectable in dental mesenchymal cells as well as dental epithelial cells. In the late stage of tooth development, the pre-odontoblasts showed strong TMSB10 expression and the pre-ameloblasts exhibited strong TMSB4X expression at approximately the same level. It was suggested that there could be a close cell-cell interaction between pre-odontoblasts and pre-ameloblasts for the formation of dentin and enamel matrix. In addition, since a strong TMSB10 signal was found in the odontoblasts in the lateral side of dental pulp and in Hertwig epithelial sheath, it is believed that the protein may be involved in the formation of the tooth root contour [32].

The protein ERP29 was isolated in a study from tooth enamel cells. It is intended primarily to normal protein secretion events such as involved in enamel secretion [33]. SLC20A2 is a membrane protein that controls the phosphate uptake during mineralization in the tooth. In the study of Merametdjian et al., the protein was studied in terms of spatio-temporal expression in murine tooth germs. The protein was strongly expressed in the postnatal stage of tooth development (2- to 10-fold higher than in other transporters) and occurred in the stratum intermedium and in the subodontoblastic cell layer of the tooth [34].

In a separate study on thymosin beta-4 (TMSB4X), it has been shown that the protein plays a key role in the odontoblastic differentiation of human tooth pulp cells and activation of the protein could provide a novel mechanism for regenerative endodontics [35].

In the study of Cai et al., the protein PDGFRB was investigated and found to have the potential of a pulp stem cell as it can generate dentin-like structures in vitro and in vivo [36].

Another study examined the protein MEPE. Obviously, the protein plays an important role in the proliferation and osteogenic differentiation of dental pulp cells [37].

The proteins extracellular serine / threonine protein kinase FAM20C, SUMO2, SUMO4, and STMN1 also play a role in odontoblastic differentiation [38–40]. Stathmin is additionally mentioned above in the study of Ma et al. on dental stem cells [25].

Using sampling of pulp with PIRL, the present study has identified several hundred proteins that were only identified in this study. The remaining proteins, which have not been mentioned, would need further investigation to finally understand their presence and task in the pulp. The next step would be to examine these proteins on diseased teeth.

The knowledge and database HPA were also used to further validate the newly identified 382 proteins (see Table 4). Each protein was examined based on its gene name. HPA is a database that shows, among other details, the distribution of proteins in human tissues and cells.

It is worth noting that the tissue of the pulp is not an item explicitly listed in HPA. HPA shows that 6.2% of the 382 proteins are identified in blood and 7.0% lymphoid tissue. Supported by the fact that the pulp, is a mixture of intercellular substances, cells, fiber elements, nerves and vessels, the above-mentioned results are assigned to the pulp tissue.

Pulp-specific proteins that have already been reported in several studies are assigned to the following tissues in HPA. TMSB4X and TMSB10 are assigned to the blood and the STMN1 to the lymphatic tissue. The proteins HMGB1, ERP29, PDGFRB, SUMO4, and SUMO2 can be found in the category of low tissue specificity. It can be hypothesized that the 32.6% of the 382 proteins that are assigned to a low tissue specificity in HPA may be proteins that are most strongly expressed in pulp tissue. MEPE and STMN1 are listed in HPA in the brain division, CD59 and TIMP2 in the placenta and SLC20A2 and FAM20C in the salivary gland. If the remaining percentage of tissue distribution of the 382 proteins is considered, most of the proteins are found in 7.7% in brain tissue, 5.4% in testis, 3.9% in liver, and 3.6% in skeletal muscles. A relatively large proportion of 8.6% are proteins not known to HPA (see Table 4). Some of these are immunoglobulins, which are part of the immune system. The other part is putative proteins. These are proteins that have an amino acid sequence that can be assigned to known proteins, but do not match in function. The remaining 25.0% is occurring in 28 other tissues in quantities from 2.4 to 0.2%. Despite the small amount, 1.5% placenta and 0.9% salivary gland specific proteins should be emphasized, since here the above-mentioned pulp-specific proteins (CD59, TIMP2, SLC20A2, FAM20C) are present.

In the study by Widbiller et al., the protein composition of human dentin was examined. Eight hundred thirteen proteins were identified [41]. Compared to our total protein list, there is matching of 176 proteins. Of the 176 proteins, even 20 proteins match our pulp protein list, which has only been identified in this study (RCN1, NUCB1, PEA15, APOC3, PTN, SKP1, ECM1, FKBP7, NUCB2, RCN3, RAB10, PTMS, APOC1, RAP1B, PGAM2, ARF5, FABP4, MEPE, ERP29, and TIMP2).

### Limitations of study

The results on the proteome of the dental pulp refer to adolescents and young adults, the typical age at which wisdom teeth are removed for dental reasons. Like other organs, the dental pulp is also subject to an aging process [42]. It is entirely possible that proteome analyses of teeth extracted from older patients have a different protein composition pattern than those from younger individuals. It should also be considered that it was a matter of the removal of functionally unstressed teeth that were exposed to the extraction trauma. Stress, especially hypoxia, causes changes in protein composition in the dental pulp [43]. In this respect, there are fundamental methodological limitations to the ex vivo examination of the dental pulp that cannot be overcome with current techniques.

## Conclusion

The aim of this study was the completion of the proteome of the human tooth pulp following the hypothesis that by using PIRL proteins are identifiable which had not been observed before by classical homogenization methods in the pulp tissue. The results of this study confirmed the initial hypothesis. Although the total number of proteins identified in this study is lower than those of some of the earlier proteomics studies of the dental pulp, a significant number of proteins were identified which had not been identified in the former studies. Twenty-eight percent of the proteins were identified in this study in addition to former studies. An overview of the molecular function, the biological processes, and the cellular component of the pulp was compiled.

## Supplementary Information

ESM 1 (XLSX 548 kb)

## References

[1] Pashley DH, Walton RE, Slavkin HC, Ingle JI, Bakland LK (2002). Histology and physiology of the dental pulp. Endodontics.

[2] Yu C, Abbott PV (2007). An overview of the dental pulp: its functions and responses to injury. Aust Dent J.

[3] Pääkkönen V, Tjäderhane L (2010). High-throughput gene and protein expression analysis in pulp biologic research: review. J Endod.

[4] Jágr M, Eckhardt A, Pataridis S, Broukal Z, Dušková J, Mikšík I (2014). Proteomics of human teeth and saliva. Physiol Res.

[5] Pääkkönen V, Ohlmeier S, Bergmann U, Larmas M, Salo T, Tjäderhane L (2005). Analysis of gene and protein expression in healthy and carious tooth pulp with cDNA microarray and two-dimensional gel electrophoresis. Eur J Oral Sci.

[6] Eckhardt A, Jágr M (2014). Proteomic analysis of human tooth pulp: proteomics of human tooth. J Endod.

[7] Eckhard U, Marino G, Abbey SR, Mathew I, Overall CM (2015). TAILS N-terminomic and proteomic datasets of healthy human dental pulp. Data Brief.

[8] Padula MP, Berry IJ, MB OR, Raymond BB, Santos J, Djordjevic SP (2017). A Comprehensive guide for performing sample preparation and top-down protein analysis. Proteomes.

[9] Zhang Y, Fonslow BR, Shan B, Baek MC, Yates JR (2013). Protein analysis by shotgun/bottom-up proteomics. Chem Rev.

[10] Lipton MS, Paša-Tolic L (2009). Mass spectrometry of proteins and peptides, methods and protocols.

[11] Schlüter H, Apweiler R, Holzhüter HG, Jungblut PR (2009) Finding one’s way in proteomics: a protein species nomenclature. Chem Cent J 9;3:11. 10.1186/1752-153X-3-11 10.1186/1752-153X-3-11 PMC2758878 19740416

[12] Tholey A, Becker A (2017). Top-down proteomics for the analysis of proteolytic events - methods, applications and perspectives. Biochim Biophys Acta, Mol Cell Res.

[13] Burden DW. Guide to the homogenization of biological samples. Random primers 2012, pp 1–25, https://opsdiagnostics.com/applications/homogenization_guide_download.html

[14] Graham, JM, Rickwood D (1997) Subcellular fractionation. Oxford. London, pp 1-28

[15] Kwiatkowski M, Wurlitzer M, Omidi M, Ren L, Kruber S, Nimer R, Robertson WD, Horst A, Miller RJ, Schlüter H (2015). Ultrafast extraction of proteins from tissues using desorption by impulsive vibrational excitation. Angew Chem Int Ed Eng.

[16] Jowett N, Wöllmer W, Mlynarek AM, Wiseman P, Segal B, Franjic K, Krötz P, Böttcher A, Knecht R, Miller RJ (2013). Heat generation during ablation of porcine skin with erbium: YAG laser vs a novel picosecond infrared laser. JAMA Otolaryngol Head Neck Surg.

[17] Franjic K, Cowan ML, Kraemer D, Miller RJ (2009). Laser selective cutting of biological tissues by impulsive heat deposition through ultrafast vibrational excitations. Opt Express.

[18] Franjic K, Miller D (2010). Vibrationally excited ultrafast thermodynamic phase transitions at the water/air interface. Phys Chem Chem Phys.

[19] Kwiatkowski M, Wurlitzer M, Krutilin A, Kiani P, Nimer R, Omidi M, Mannaa A, Bussmann T, Bartkowiak K, Kruber S, Uschold S, Steffen P, Lübberstedt J, Küpker N, Petersen H, Knecht R, Hansen NO, Zarrine-Afsar A, Robertson WD, Miller RJD, Schlüter H (2016). Homogenization of tissues via picosecond-infrared laser (PIRL) ablation: giving a closer view on the in-vivo composition of protein species as compared to mechanical homogenization. J Proteome.

[20] Mi H, Muruganujan A, Ebert D, Huang X, Thomas PD (2019). PANTHER version 14: more genomes, a new PANTHER GO-slim and improvements in enrichment analysis tools. Nucleic Acids Res.

[21] Hulsen T, de Vlieg J, Alkema W (2008). BioVenn - a web application for the comparison and visualization of biological lists using area-proportional Venn diagrams. BMC Genomics.

[22] UniProt: the universal protein knowledge base. Nucleic Acids Res. 2017 45: D158D169, https://www.uniprot.org/. Accessed 2020 10.1093/nar/gkw1099 PMC5210571 27899622

[23] Koscielny G, An P, Carvalho-Silva D, Cham JA, Fumis L, Gasparyan R, Hasan S, Karamanis N, Maguire M, Papa E, Pierleoni A, Pignatelli M, Platt T, Rowland F, Wankar P, Bento AP, Burdett T, Fabregat A, Forbes S, Gaulton A, Gonzalez CY, Hermjakob H, Hersey A, Jupe S, Kafkas Ş, Keays M, Leroy C, Lopez FJ, Magarinos MP, Malone J, McEntyre J, Munoz-Pomer Fuentes A, O'Donovan C, Papatheodorou I, Parkinson H, Palka B, Paschall J, Petryszak R, Pratanwanich N, Sarntivijal S, Saunders G, Sidiropoulos K, Smith T, Sondka Z, Stegle O, Tang YA, Turner E, Vaughan B, Vrousgou O, Watkins X, Martin MJ, Sanseau P, Vamathevan J, Birney E, Barrett J, Dunham I (2017). Open Targets: a platform for therapeutic target identification and validation. Nucleic Acids Res.

[24] The Human Protein Atlas, https://www.proteinatlas.org. Accessed 2020

[25] Ma D, Cui L, Gao J, Yan W, Liu Y, Xu S, Wu B (2014). Proteomic analysis of mesenchymal stem cells from normal and deep carious dental pulp. PLoS One.

[26] Jágr M, Eckhardt A, Pataridis S, Foltán R, Myšák J, Mikšík I (2016). Proteomic analysis of human tooth pulp proteomes - comparison of caries-resistant and caries-susceptible persons. J Proteome.

[27] McClanahan SB, Turner DW, Kaminski EJ, Osetek EM, Heuer MA (1991). Natural modifiers of the inflammatory process in the human dental pulp. J Endod.

[28] Telkoparan P, Erkek S, Yaman E, Alotaibi H, Bayık D, Tazebay UH (2013). Coiled-coil domain containing protein 124 is a novel centrosome and midbody protein that interacts with the Ras-guanine nucleotide exchange factor 1B and is involved in cytokinesis. PLoS One.

[29] Zhang X, Jiang H, Gong Q, Fan C, Huang Y, Ling J (2014). Expression of high mobility group box 1 in inflamed dental pulp and its chemotactic effect on dental pulp cells. Biochem Biophys Res Commun.

[30] Accorsi-Mendonça T, Silva EJ, Marcaccini AM, Gerlach RF, Duarte KM, Pardo AP, Line SR, Zaia AA (2013). Evaluation of gelatinases, tissue inhibitor of matrix metalloproteinase-2, and myeloperoxidase protein in healthy and inflamed human dental pulp tissue. J Endod.

[31] Jeanneau C, Rufas P, Rombouts C, Giraud T, Dejou J, About I (2015). Can pulp fibroblasts kill cariogenic bacteria? Role of complement activation. J Dent Res.

[32] Shiotsuka M, Wada H, Kiyoshima T, Nagata K, Fujiwara H, Kihara M, Hasegawa K, Someya H, Takahashi I, Sakai H (2013). The expression and function of thymosin beta 10 in tooth germ development. Int J Dev Biol.

[33] Hubbard MJ, McHugh NJ, Carne DL (2000). Isolation of ERp29, a novel endoplasmic reticulum protein, from rat enamel cells. Evidence for a unique role in secretory-protein synthesis. Eur J Biochem.

[34] Merametdjian L, Beck-Cormier S, Bon N, Couasnay G, Sourice S, Guicheux J, Gaucher C, Beck L (2018). Expression of phosphate transporters during dental mineralization. J Dent Res.

[35] Lee SI, Kim DS, Lee HJ, Cha HJ, Kim EC (2013). The role of thymosin beta 4 on odontogenic differentiation in human dental pulp cells. PLoS One.

[36] Cai S, Zhang W, Chen W (2016). PDGFRβ(+)/c-kit(+) pulp cells are odontoblastic progenitors capable of producing dentin-like structure in vitro and in vivo. BMC Oral Health.

[37] Wei X, Liu L, Zhou X, Zhang F, Ling J (2012). The effect of matrix extracellular phosphoglycoprotein and its downstream osteogenesis-related gene expression on the proliferation and differentiation of human dental pulp cells. J Endod.

[38] Li Q, Yi B, Feng Z, Meng R, Tian C, Xu Q (2018). FAM20C could be targeted by TET1 to promote odontoblastic differentiation potential of human dental pulp cells. Cell Prolif.

[39] Hosoya A, Yukita A, Ninomiya T, Hiraga T, Yoshiba K, Yoshiba N, Kasahara E, Nakamura H (2013). Localization of SUMOylation factors and Osterix in odontoblast lineage cells during dentin formation and regeneration. Histochem Cell Biol.

[40] Zhang X, Ning T, Wang H, Xu S, Yu H, Luo X, Hao C, Wu B, Ma D (2019). Stathmin regulates the proliferation and odontoblastic/osteogenic differentiation of human dental pulp stem cells through Wnt/β-catenin signaling pathway. J Proteome.

[41] Widbiller M, Schweikl H, Bruckmann A, Rosendahl A, Hochmuth E, Lindner SR, Buchalla W, Galler KM (2019). Shotgun proteomics of human dentin with different prefractionation methods. Sci Rep.

[42] Maeda H (2020). Aging and senescence of dental pulp and hard tissues of the tooth. Front Cell Dev Biol.

[43] Rombouts C, Giraud T, Jeanneau C, About I (2017). Pulp vascularization during tooth development, regeneration, and therapy. J Dent Res.

